# Treatment outcomes and costs of a simplified antiviral treatment strategy for hepatitis C among monoinfected and HIV and/or hepatitis B virus‐co‐infected patients in Myanmar

**DOI:** 10.1111/jvh.13405

**Published:** 2020-10-09

**Authors:** Yin Min Thaung, Charles S. Chasela, Kara W. Chew, Thomas Minior, Aye A. Lwin, Yi Y. Sein, Ndeye Drame, Fadzai Marange, Charles van der Horst, Hnin T. Thwin, Morgan J Freiman, Malini M. Gandhi, Murdo Bijl, Constance Wose Kinge, Sydney Rosen, Si Thura, Sofiane Mohamed, Thembisile Xulu, Aung Y. Naing, Matthiue Barralon, Clint Cavenaugh, Khin P. Kyi, Ian Sanne, for EQUIP Health

**Affiliations:** ^1^ Community Partners International Yangon Myanmar; ^2^ Right to Care/EQUIP Health Pretoria South Africa; ^3^ Department of Epidemiology and Biostatistics School of Public Health Faculty of Health Sciences University of the Witwatersrand Johannesburg South Africa; ^4^ David Geffen School of Medicine at UCLA Los Angeles CA USA; ^5^ U.S. Agency for International Development Washington DC USA; ^6^ Advanced Biological Laboratories (ABL) SA Rue des jardiniers Luxembourg; ^7^ Myanmar Liver Foundation Yangon Myanmar; ^8^ School of Public Health Boston University Boston MA USA; ^9^ University of North Carolina Chapel Hill NC USA; ^10^ Boston University School of Medicine Boston MA USA; ^11^ Asian Harm Reduction Network Kachin Myanmar; ^12^ Division of Epidemiology and Surveillance National Institute for Occupational Health Johannesburg South Africa; ^13^ Hepatitis Virus Diversity Research Unit Department of Internal Medicine School of Clinical Medicine Faculty of Health Sciences University of the Witwatersrand Johannesburg South Africa; ^14^ Faculty of Health Sciences University of the Witwatersrand Johannesburg South Africa; ^15^ Department of Medicine Faculty of Health Sciences University of the Witwatersrand Johannesburg; ^16^ Immunology Research Division, Department of Medical Research Ministry of Health and Sports Yangon Myanmar

**Keywords:** HBV, HCV, HIV, people who inject drugs, sustained virologic response

## Abstract

Access to hepatitis C virus (HCV) testing and treatment is limited in Myanmar. We assessed an integrated HIV and viral hepatitis testing and HCV treatment strategy. Sofosbuvir/velpatasvir (SOF/VEL) ± weight‐based ribavirin for 12 weeks was provided at three treatment sites in Myanmar and sustained virologic response (SVR) assessed at 12 weeks after treatment. Participants co‐infected with HBV were treated concurrently with tenofovir. Cost estimates in 2018 USD were made at Yangon and Mandalay using standard micro‐costing methods.

803 participants initiated SOF/VEL; 4.8% were lost to follow‐up. SVR was achieved in 680/803 (84.6%) by intention‐to‐treat analysis. SVR amongst people who inject drugs (PWID) was 79.7% (381/497), but 92.5% among PWID on opioid substitution therapy (OST) (74/80), and 97.4% among non‐PWID (298/306). Utilizing data from 492 participants, of whom 93% achieved SVR, the estimated average cost of treatment per patient initiated was $1030 (of which 54% were medication costs), with a production cost per successful outcome (SVR) of $1109 and real‐world estimate of $1250. High SVR rates were achieved for non‐PWID and PWID on OST. However, the estimated average cost of the intervention (under the assumption of no genotype testing and reduced real‐world effectiveness) of $1250/patient is unaffordable for a national elimination strategy. Reductions in the cost of antivirals and linkage to social and behavioural health services including substance use disorder treatment to increase retention and adherence to treatment are critical to HCV elimination in this population.

## 1 INTRODUCTION

Hepatitis C virus (HCV) infection is a deadly but curable disease that disproportionately affects people in low‐ and middle‐income countries (LMICs).^1^, ^2^, ^3^ It is common among people who inject drugs (PWID) and even in otherwise low prevalence settings.^2^, ^4^ Chronic HCV infection is expected in 55% to 85% of untreated cases and is associated with liver cirrhosis, liver failure, hepatocellular carcinoma and death. Despite the development of highly effective direct‐acting antiviral (DAA) treatment, which can cure HCV infection with 8‐12 weeks of therapy, HCV remains a leading cause of mortality worldwide, causing more than 350,000 deaths each year.^3^, ^5^

The WHO’s Global Health Sector Strategy sets goals of a 90% reduction in new HCV infections and a 65% reduction in HCV‐related mortality by 2030.^6^ Strategies for achieving these targets include scaling up access to affordable testing and treatment.^7^ Progress in HCV treatment scale‐up is encouraging, with more than 3 million treated globally with DAAs since 2015 but testing coverage and diagnosis rates are still less than 10% in LMICs.^3^

In Myanmar, the prevalence of HCV infection is estimated at 2.7%,^8^ HCV antibody positivity among PWID at 48.1%, and treatment access in the region under 1%.^9^ HCV infection is estimated to account for 25% of hepatocellular carcinoma.^4^ Myanmar's HIV epidemic ranks among the most serious in Asia and is concentrated among men who have sex with men (MSM), PWID and female sex workers (FSW).^5^, ^10^, ^11^ HCV/HIV co‐infection rates vary, with estimates ranging from 5% to 22.8% and high risk among PWID.^10^, ^12^, ^13^, ^14^

Mynamar has a national treatment and testing strategy for the elimination of HCV. Availability and access to HCV testing and treatment, fear of prosecution for drug use, and high stigma for key populations, results in most presenting for HCV and HIV treatment are already well advanced in their illness. EQUIP launched a single arm demonstration project to evaluate an integrated, simplified protocol for testing and treating HCV and HIV among key populations in Myanmar. We describe the treatment outcomes and estimated costs for simplified HCV testing and DAA treatment with SOF/VEL with or without RBV for patients with and without HIV co‐infection.

## 2 METHODS

### 2.1 Study sites and population

Enrolment occured between December 2017 and November 2018 at three clinical facilities in Myanmar: the Than Sitt Charity Clinics in Yangon and Mandalay, both operated by the Myanmar Liver Foundation (MLF), and the Asian Harm Reduction Network (AHRN) Clinic in Waimaw, Kachin. Patients were referred by peers, community workers, civil society groups, the General Practitioners Society, the National AIDS Program (NAP), the Myanmar Anti‐Narcotic Association (MANA), Medicins Sans Frontier Holland (MSFH) and Médecins du Monde (MdM). The target populations were PLHIV, PWID, MSM and FSW, although initial enrolment included populations outside of these, most of whom could not afford private treatment.

### 2.2 Selection criteria

Eligible participants were HCV viremic, HCV treatment naïve or experienced (prior pegylated interferon [PegIFN] and RBV only), and 18 years or older, with HCV genotype 1, 2, 3, 4, 5 or 6, with or without HIV‐1 co‐infection. Patients with compensated cirrhosis (Child‐Pugh Class A) and HBV infection were eligible; those with decompensated cirrhosis (Child‐Pugh Class B or C) or prior treatment with HCV DAAs were not eligible. Patients who were ineligible for HCV treatment were referred to other treatment centres. All participants provided written informed consent.

### 2.3 Intervention description

The intervention combined HCV and HIV testing, simplified HCV treatment, and HIV treatment initiation for those with HIV co‐infection not yet on antiretroviral therapy (ART). HCV treatment was with fixed‐dose combination sofosbuvir 400 mg/velpatasvir 100 mg (SOF/VEL) ± weight‐based ribavirin for 12 weeks. Ribavirin was initially included in the regimen for genotype 3 participants who were cirrhotic or who had previously failed an interferon‐based treatment regimen. In April 2018, 5 months into enrolment, based on more recent findings on treatment response rates with SOF/VEL without ribavirin for genotype 3 infection, including amongst PWID and in resource‐limited settings in Asia,^15^, ^16^, ^17^, ^18^ the protocol was revised to treat all participants with SOF/VEL alone, for 12 weeks, regardless of genotype. As such, genotyping was not required prior to treatment initiation. This allowed the inclusion of the third site in rural Kachin called Waimaw, which faced barriers to laboratory testing and had limited resources to monitor for ribavirin‐associated and other toxicities.

**FIGURE 1:**
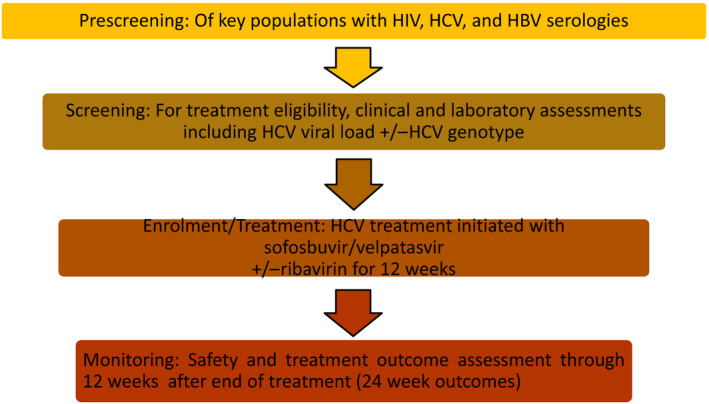
Intervention procedures

Participants with hepatitis B surface antigen positivity were concurrently treated with tenofovir disoproxil fumarate (TDF). Provision of HIV and hepatitis B treatment was derived from national or USAID supported HIV treatment programmes. The first‐line HIV treatment regimen in Myanmar—TDF/lamivudine (3TC)/efavirenz—posed a challenge as efavirenz could not be co‐administered with velpatasvir due to drug‐drug interactions. As such, the NAP supported the substitution of dolutegravir or another alternative (such as lopinavir/ritonavir) for efavirenz. Participants were followed for 24 weeks (through 12 weeks after treatment completion), including assessments of HCV treatment outcomes and safety. Intervention steps are illustrated in Figure 1.

### 2.4 Clinical and laboratory evaluations

#### 2.4.1 Clinical evaluations and drug dispensing

Medical history and clinical assessment were undertaken at baseline and repeated at week 24; additional clinical assessments were performed at clinicians’ discretion. Concomitant medications were recorded. Liver disease stage was defined based on either ultrasound (liver imaging consistent with cirrhosis [surface nodularity, heterogenous, course echotexture and enlarged caudate lobe]), AST‐to‐platelet ratio index (APRI) score ≥2.0, or Fibroscan ≥12.5 kPa. For cirrhotic patients, Child‐Pugh score was calculated; compensated cirrhosis was defined as Child‐Pugh Score ≤6. SOF/VEL and TDF were dispensed at weeks 0, 4 and 8. Ribavirin was dispensed at weeks 0, 2, 4 and 8. A pharmacist provided drug information at each visit. Counselling was provided at baseline and weeks 4, 8, 12 and 24 by a social worker, or peer counsellor on study procedures and treatment adherence, HCV transmission and re‐infection risks and harm reduction, with referral to harm reduction services as appropriate for substance use comorbidities and HIV and HCV risk factors.

#### 2.4.2 Laboratory evaluations

The screening laboratory evaluations included serum HCV antibody if not already available in the medical record, haemoglobin, platelets, AST, ALT, total bilirubin, albumin, creatinine, blood urea nitrogen, pregnancy testing, prothrombin time/International Normalized Ratio (INR), point‐of‐care (POC) HBV serologies with WHO qualified SD Bioline (HBcAb, HBsAg, HBsAb), POC rapid HIV testing (Determine, Unigold and Statpak) and CD4^+^ T‐cell count for HIV‐infected participants. Laboratory results were also obtained from the medical record if reported within the specified window (namely HCV RNA at any time prior to study entry, HIV testing at any time prior to entry if positive, and CD4^+^ T‐cell count within 90 days). HCV RNA testing was undertaken for all participants with positive HCV antibody results, as described below. HCV genotyping, subtyping and resistance testing for all HCV viremic patients were performed at the Department of Medical Research, Ministry of Health (Yangon) using the Advanced Biological Laboratories (ABL) UltraGene‐HCV assay on Thermofisher QuanStudio 3 qPCR system and resistance was performed using the ABL DeepChek assay on the Thermofisher SeqStudio sequencer. All participants who initiated HCV treatment had the following laboratory tests at week 24: HCV RNA, haemoglobin, platelets, AST, ALT, bilirubin, albumin, creatinine, blood urea nitrogen, prothrombin time/INR and HIV RNA (if HIV‐infected). Participants receiving ribavirin additionally had haemoglobin monitoring at weeks 2, 4 and 8. Pregnancy testing was required for all females of childbearing potential at screening and at weeks 4, 8, 12 and 24 for those assigned to ribavirin. Participants with eGFR < 60 at screening and also receiving tenofovir had creatinine monitoring at weeks 4 and 12. Participants with isolated hepatitis B core Ab positivity (HBcAb+/HBsAg‐/HBsAb‐) had hepatic liver tests (AST, ALT, bilirubin) at weeks 4, 8 and 12 to assess for potential HBV reactivation.

#### 2.4.3 Measurement of HCV viral load

HCV viral load was measured using near POC Cepheid Xpert^®^ HCV Viral Load (Cepheid, CA, USA) and/or Roche (COBAS^®^AmpliPrep/COBAS ^®^TaqMan ^®^HCV quantitative) assay. Xpert^®^ and Roche HCV viral load was performed at Ni‐Ni Laboratory in Yangon for the Yangon and Mandalay sites. Xpert^®^ HCV viral load was performed on site at AHRN Waimaw clinic, Kachin. Roche HCV viral load estimation served as the standard of care VL assay prior to validation of Xpert^®^ HCV VL. The limits of quantification were 10 IU/mL for Xpert, and 15 IU/mL for Xpert^®^ and Roche assays respectively.

### 2.5 HCV Genotyping

HCV genotyping and subtyping were performed at the Department of Medical Research, Myanmar Ministry of Health (Yangon) using Advanced Biological Laboratories (ABL) UltraGene‐HCV assay (lower limit of detection of 20 IU/mL) and DeepChek‐HCV genotyping assay (ABL SA) on Thermofisher QuanStudio 3 qPCR and ThermoFisher SeqStudio sequencer systems. The DeepChek‐HCV genotyping assay differentiates all six HCV genotypes and can discriminate HCV 1b/2k chimeras and subtypes 1a and 1b. After RNA extraction from plasma and RT‐PCR of the HCV genome according to the manufacturer's instructions, amplicons were obtained for 5’UTR, NS5B and NS5A regions. DNA products were sequenced with specific primers by capillary electrophoresis according to the manufacturer's instructions. Sanger sequencing, the BigDye X Terminator (Life Technologies, Carlsbad, California, USA) were used.

The sequences obtained were read and aligned with the DeepChek‐HCV. A consensus sequence was generated for each sample and compared with the HCV genomic Basic Local Alignment Search Tool (BLAST) bank for determination of the HCV genotype and subtype. A threshold for similarity of minimum 85% was used to consider the genotype or subtype.^19^, ^20^

### 2.6 Outcomes

The two primary outcomes of the analysis were sustained virologic response (SVR) at 12 weeks after the end of treatment and cost per patient with SVR. Other outcomes included safety (adverse events) during the treatment period. For the cost analysis, we assigned each patient one of four outcomes at 24 weeks after treatment initiation: (a) treatment success (SVR 12 weeks after therapy completion); (b) treatment failure (HCV viraemia greater than the lower limit of detection 12 weeks after therapy completion); (c) loss to follow‐up (did not return to clinic for 24‐week evaluation); or (d) death (died within the 24‐week study period).

### 2.7 Data analysis

#### 2.7.1 Outcomes

Baseline sociodemographic, clinical and laboratory characteristics of the screened participants were calculated using proportions or means (standard deviations) or medians (interquartile ranges) for continuous data. The proportion with SVR was calculated for each clinical site and overall, by intention‐to‐treat (ITT). Differences between those successfully treated SVR achieved compared to those who failed treatment were assessed by t test or chi‐square test as appropriate, and by multivariable logistic regression. A *P*‐level of less than .2 in the bivariate analysis was used to select variables for multivariable analysis. A final model was determined using stepwise backward elimination method and only *P*‐level of less than .05 was considered for the final model. Multi collinearity test, specification error testing and Hosmer‐Lemeshow test (HL test) for logistic regression were undertaken on the final model. Data were analysed using STATA version 15 SE (StataCorp 2017. Stata Statistical Software: Release 15. College Station, TX: StataCorp LLC).

##### Costs

Cost estimates were made at two sites, Yangon and Mandalay, using data from the first 492 patients from these sites. Costs were estimated from the provider perspective from initial screening date until assessment of outcome using standard economic methods described previously.^21^, ^22^ We determined variable patient resource utilization from study case reporting forms and estimated resource utilization from average clinic site capacity and total annual visits. We then multiplied the quantity of each resource used by each patient by the associated unit cost to determine a total cost per patient.

Resources incurring costs included events, laboratory assessments, HCV and HBV medications, clinic staff, indirect costs, education and outreach during the 24‐week follow‐up period. Events included physical examinations (physician visit), counselling visits and Fibroscans (supplies only). Laboratory assessments included HCV RNA, HCV genotyping, HIV and HBV testing, pregnancy testing, PT/INR, and blood counts and chemistries. Indirect costs included support staff/personnel, building costs, equipment and office supplies. HIV medications were utilized but were paid for separately by the government HIV programme and are excluded from the cost analysis. Equipment and technicians’ time for Fibroscan^®^ testing were donated and are also excluded. Costs attributable to the research study but unlikely to be incurred in routine implementation (research staff, education and outreach activities, tariffs and shipping charges for supplies) were included only in Scenario 2.

For the cost analysis, we considered four scenarios: (a) observed costs excluding research‐related expenses (proxy for real‐world setting); (b) observed costs including research expenses; (c) observed costs excluding research related expenses and excluding routine HCV genotype testing; and (d) observed costs excluding research‐related expenses and increasing the treatment failure rate to 14% to simulate potential poorer treatment adherence and/or effectiveness in a real‐world setting.

We also evaluated the average resource utilization per patient by cost category and HCV treatment outcome and calculated 95% confidence intervals by outcome category. We calculated the production cost of a successful outcome by dividing the sum of all costs by the number of successful outcomes. We applied the average exchange rate for 2018 of 1.00 United States dollar (USD) = 1547.71 Myanmar Kyat (MMK).

### 2.8 Ethical considerations

The study was reviewed by Myanmar Ministry of Health and Sports Institutional Technical and Ethical Review Board, University of Public Health (ITERB‐2017/Research/18), the University of the Witwatersrand Human Research Ethics Committee (M17078) and the UCLA Medical Institutional Review Board (#18‐00003). The Boston University Institutional Review Board approved analysis of a de‐identified analytic dataset (H‐37820). All patients provided written informed consent. The study is registered at ClinicalTrials.gov (NCT03579576).

## 3 RESULTS

### 3.1 Enrolment and patient characteristics

**FIGURE 2:**
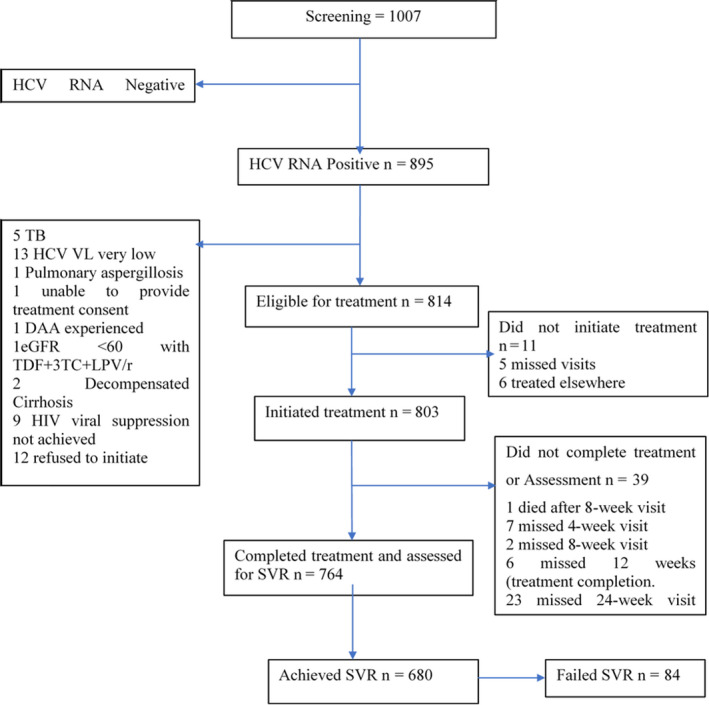
Study enrolment and retention

We screened 1007 HCV antibody positive patients and enrolled those eligible at the three study sites between 18th December 2017 and 11th November. We excluded 193 of those screened for the reasons listed in Figure 2, including 112 who did not have detectable HCV RNA. A total of 814 (80.8%) patients were eligible for treatment, 803 initiated treatment and 764 (95%) completed 24 weeks of follow‐up. Out of those screened, 46.8% (444) and 46.7% (443) were HCV monoinfected and HCV/HIV co‐infected respectively with the remaining 6.5% HCV/HBV and HCV/HIV/HBV infected. Most HCV monoinfected were in Yangon while most HCV/HIV co‐infected were screened in Kachin. Out of the 1007 screened participants, 657 were PWID and 402 (61.2%) of these were recruited from Kachin. Most patients assessed were not cirrhotic (82.0%); 17.8%, and 0.2% had compensated and decompensated cirrhosis, respectively (Table 1).

**TABLE 1:** Patient characteristics at screening, by treatment site

Characteristic	Yangon (n = 427)	Mandalay (n = 168)	Kachin (n = 412)	Total (N = 1007)
Age (y) (median, interquartile range)	43 (18‐70)	35 (19‐71)	31 (18‐65)	36 (18‐71)
Sex(%): Female	158 (37.0)	17 (10.1)	13 (3.2)	188 (18.7)
Education (%): beyond primary	359 (84.1)	152 (90.5)	351 (85.2)	862 (85.6)
Risk Groups (%)				
MSM	1 (0.2)	0	0	1 (0.1)
General population	247 (57.9)	22 (13.1)	0 (0.0)	269 (26.7)
PLHIV	15 (3.5)	22 (13.1)	9 (2.2)	46 (4.6)
PLHIV/MSM	4 (0.9)	1 (0.6)	0	5 (0.5)
PLHIV sexual partner	1 (0.2)	0	0	1 (0.1)
PLHIV sexual partner of HCV+	1 (0.2)	0	1 (0.2)	
PWID	49 (11.5)	29 (17.3)	3 (0.7)	81 (8.0)
PWID/MAT	46 (10.8)	47 (28.0)	54 (13.1)	147 (14.6)
PWID/MSM	1 (0.23)	0	0	1 (0.1)
PWID/MSM/MAT	1 (0.23)	1 (0.6)	0	2 (0.2)
PWID/PLHIV	17 (4.0)	22 (13.1)	221 (53.6)	260 (25.8)
PWID/PLHIV/MAT	10 (2.3)	24 (14.3)	123 (29.9)	157 (15.6)
PWID/PLHIV/MSM	1 (0.2)	0	0	1 (0.1)
PWID/PLHIV/MSM/MAT	3 (0.7)	0	0	3 (0.3)
PWID/PLHIV/SW	0	0	1 (0.2)	1 (0.1)
PWID/sexual partner of HCV+/ MAT	4 (0.9)	0	0	4 (0.4)
SW	1 (0.2)	0	0	1 (0.1)
Sexual partner of HCV+	25 (5.9))	0	0	25 (2.5)
Total PWID	132 (30.9)	123 (73.2)	402 (97.6)	657 (65.2)
PWID on MAT	64 (48.5)	72 (58.5)	177 (44.0)	313 (47.6)
Marital status: ever married (%)	285 (66.7)	95 (56.6)	218 (52.9)	598 (59.4)
Median BMI (kg/m^2^) (IQR)	22.4 (20.2‐25.2)	20.1 (18.2‐22.9)	20.3 (18.7‐21.9)	20.9 (19.1‐23.5)
Cirrhosis %				
Compensated	114 (28.0)	16 (9.6)	27 (8.6)	157 (17.8)
Decompensated	0	0	2 (0.6)	2 (0.2)
No cirrhosis	293 (72.0)	145 (90.1)	284 (90.7)	722 (82.0)
Infection status (%)				
HCV monoinfected	319 (81.0)	82 (54.3)	43 (10.7)	444 (46.8)
HCV/HIV	50 (12.7)	65 (43.1)	328 (81.4)	443 (46.7)
HCV/HBV	23 (5.8)	0 (0)	5 (1.2)	28 (3.0)
HCV/HIV/HBV	2 (0.5)	4 (2.7)	27 (6.7)	33 (3.5)
Genotype (subtype)				
1	38 (11.5)	14 (13.7)	6 (2.8)	58 (9.0)
2	4 (1.2)	0	0	4 (0.6)
3	157 (47.4)	42 (41.2)	113 (53.3)	312 (48.4)
4	2 (0.6)	1 (1.0)		3 (0.5)
6	129 (39.0)	45 (44.1)	93 (43.9)	267 (41.4)
Mixed	1 (0.3)			1 (0.2)
HCV RNA, Log_10_IU/mL (median, IQR)	8.4 (5.8‐6.1)	6.3 (5.5‐6.8)	6.4 (5.6‐6.7)	7.1 (5.7‐7.0)
Haemoglobin, g/dL (median IQR)	12.6 (13.6‐14.6)	14.3 (13.1‐15.8)	13.8 (13‐14.7)	13.9 (12.8‐14.9)
Creatinine, mg/dL (median, IQR)	0.8 (0.9‐1.0)	0.9 (0.7‐1.0)	0.81 (0.72 0.92	0.8 (0.72‐1.0)
ALT, U/L (median, IQR)	43 (29‐65)	49 (34‐97)	44 (30.4‐64.5)	45 (30‐67)
Bilirubin, mg/dL (median, IQR)	0.5 (0.4‐0.7)	0.5 (0.4‐0.7)	0.46 (0.32‐0.61)	5 (0.37‐0.7)
Albumin, g/dL (median, IQR)	4.4 (4.2‐4.6)	4.4 (–4.1‐4.6)	4.1 (3.8‐4.3))	4.3 (4‐4.5)
INR (median IQR)	0.86 ( 0.81‐0.9)	0.9 (0.8‐0.9)	1.2 (1.2‐1.3)	0.91 (0.8‐1.2)
Platelets (×10^9^/L) (median (IQR)	248 (204‐294)	240 (185‐280)	182 (132‐245)	226 (168‐275)
APRI Score (median IQR)	0.41 (0.29‐0.71)	0.56 (0.36‐1.0)	0.71 (0.42‐1.2)	0.54 (0.33‐0.90)

Note

HBV, hepatitis B virus; HCV, hepatitis C virus; INR, International Normalized Ratio; MAT, medication‐assisted treatment; MSM, men who have sex with men; Partner +, sexual partner of HCV infected person; PLHIV, people living with human immunodeficiency virus (HIV) infection; PWID, people who inject drugs; SW, sex worker.

Six‐hundred and eighty participants had HCV genotyping results available at the time of this analysis. Of these, the most common genotypes were 3 (48.8%) and 6 (41.4%). The subtypes were mostly 3b (36%) followed by 6n (19%) and many new genotype 6 subtypes were found in Myanmar.

### 3.2 Treatment outcomes

Treatment outcomes by site are reported in Table 2. Of the 803 patients who initiated HCV treatment, 12 were treated with ribavirin. There were three adverse events, and none was related to treatment. Two of them were serious adverse events, neither of which was related to study treatment: one with hospitalization for low grade fever and hypokalaemia at week 4 on study that was treated and resolved, one death due to tuberculosis at week 12, and a diabetic foot infection at week 24, that was treated and resolved.

**TABLE 2:** Study outcomes at 24 weeks after study enrolment, by site

Outcome	Yangon	Mandalay	Kachin	Total
		N (%)		
Treatment success (SVR)	347 (96)	110 (83)	223 (72)	680 (85)
Treatment failure	8 (2)	9 (7)	67 (22)	84 (10)
Lost to follow‐up after treatment initiation	4 (1)	13 (10)	21 (7)	38 (5)
Died	1 (0)	0 (0)	0 (0)	1 (0)
Total	360 (100)	132 (100)	311 (100)	803 (100)

After treatment initiation, 39 participants were counted as lost to follow‐up (LTFU), 1 died, and 23 were known to have completed treatment but missed the week 24 SVR assessment. A total of 764 participants were assessed for SVR at 24 weeks. By intention‐to‐treat analysis (ITT), 123 failed (HCV viraemia above the limit of quantification at week 24), for an overall treatment success rate of 680/803 (85%). SVR rates differed significantly by site, risk group, and HIV co‐infection status, with the greatest number of treatment failures and losses to follow‐up in Kachin. Treatment failure was greater among PWID than other groups. Out of 803 who initiated treatment, 61.9% (497) were PWID and of the PWID assessed at 24 weeks, 76.7%. achieved SVR. Amongst PWID who initiated HCV treatment, 80 were on opioid substitution therapy (OST) at treatment initiation; those on OST were more likely to achieve SVR by ITT: 92.5% vs 73.6% among PWID not on OST.

### 3.3 Predictors of SVR

In Table 3, characteristics of participants are compared between the SVR group and the non‐SVR group by ITT population. ITT univariate regression showed significant differences in the odds of achieving a successful treatment response by treatment site, age, PWID status, gender, BMI, genotype 3, HIV co‐infection and INR. In multivariate analysis, the odds of failure were 3.4 times higher among PWID (AOR 3.4 (CI: 1.2‐9.3)) and odds to fail was reduced by 80% among PWIDS on OST (AOR 0.2 (CI: 0.1‐0.6)) and those treated in Yangon (AOR 0.2 (CI: 0.1‐0.5)). HIV co‐infection, genotype 3 and sex were no longer significant in multivariable analysis. The final model showed no evidence of specification error (hatsq, *P* = .674; hat, *P* = .47), no evidence of multicollinearity (mean variance inflation factor ‐VIF = 1.5) and an overall good fit (Hosmer‐Lemeshow goodness‐of‐fit test, *P* = .8451).

**TABLE 3:** Factors influencing treatment response (intention‐to‐treat, N = 803)

Characteristic	Treatment failure (n = 123)	Treatment success (n = 680)	ITT (N = 803)	OR (95%CI) for treatment failure	*P*‐value	AOR (95% CI) for treatment failure	*P*‐value^a^
Region							
MLF mandalay	22 (17.7)	110 (16.2)	132 (16.4)	(ref)			
Kachin	89 (71.8)	222 (32.7)	311 (38.7)	2.0 (1.2‐3.4)	<0.009	1.4 (0.7‐2.6)	
MLF Yangon	13 (10.5)	347 (51.1)	360 (44.8)	0.2 (0.1‐0.0.4)	< 0.001	0.2 (0.1‐0.5)	0.001
Age (mean, SD)	32 (9.5)	39 (11.6)	38.2 (11.6)	0.9 (0.9‐1.0)	<0.001	1.0 (0.9‐1)	0.032
Sex (%)							
Female	2 (1.6)	161 (23.7)	163 (20.3)	0.1 (0.01‐0.2)	<0.001		
Male	122 (98.4)	518 (76.3)	640 (79.7)	1.00 (ref)			
Education							
Primary and below	21 (16.9)	94 (13.9)	115 (14.3)				
Beyond primary	103 (83.1)	585 (86.2)	688 (85.7)	0.8 (0.5‐1.3)	0.367		
PWID (%)							
PWID	116 (93.5)	381 (56)	497 (61.9))	11.3 (5.5‐23.6)	<0.001	3.4 (1.2‐9.3)	0.020
Non‐PWID	8 (6.5)	98 (44)	306 (38.1)	1.000 (ref)			
PWIDs (OST) (%)	6 (7.5)	74 (92.5)	80 (100)	0.2 (0.1‐0.5)	0.001	0.2 (0.1‐0.6)	0.002
PWIDS (No OST) (%)	110 (26.4)	307 (73.6)	417 (100)	1.00 (ref)			
Marital status (%)							
Single	70 (53.23)	253 (36.4)	323 (38.9)	1.00 (ref)			
Ever married	54 (46.77)	426 (63.6)	480 (61.0)	0.5 (0.3‐0.7)	<0.001		
Mean BMI (kg/m^2^) (SD)	20.3 (2.9)	21.8 (3.9)	21.6 (3.8)	0.9 (0.8‐0.9)	<0.001		
Cirrhosis %							
Compensated	10 (8.1)	130 (19.2)	140 (17.4)	1.00 (ref)			
No cirrhosis	114 (91.9)	549 (80.9)	663 (82.6)	2.7 (1.4‐5.3)	0.004		
Infection status (%)							
HCV monoinfected	20 (16.1)	395 (58.2)	415 (51.7)	1.00 (ref)			
HCV/HIV	98 (79.0)	253 (37.26)	351 (43.71)	7.7 (4.6‐12.7)	<0.001		
HCV/HBV	0	20 (2.95)	20 (2.5)	1.00 ( empty)			
HCV/HIV/HBV	6 (4.84)	11 (1.62)	17 (2.1)	10.8 (3.6‐32.1)	<0.001		
Genotype (subtype)							
1	3 (3.1)	55 (10.15)	58 (9.08)	1.00 (ref)			
2	0	4 (0.74)	4 (0.63)	1.00 (empty)			
3 ( 3a/3b)	53 (54.6)	256 (47.23)	309 (48.36)	3.8 (1.1‐12.6)	0.029		
4	0	3 (0.6)	3 (0.47)	1.00 (empty)			
6	41 (42.3)	224 (41.3)	265 (41.47)	3.4 (1.0‐11.2)	0.050		
Mixed	0	0	0	0			
Haemoglobin, g/dL (median, IQR)	13.9 (10‐7.2)	13.9 (5.3‐21.4)	13.9 (5.3‐21.4)	1.0 (0.9‐1.1)	0.962		
Log10 viral load (median, IQR)				0.8 (0.7‐0.9)	<0.001		
Creatinine, mg/dL (median, IQR)	0.82 (0.5‐1.4)	0.84 (0.28‐1.8)	0.83 (0.28‐1.8)	0.5 (0.2‐1.5)	0.203		
ALT U/L (median, IQR)	40.9 (30.5‐69)	45 (30‐67)	44 (30‐67)	1.00 (0.9‐1.0)	0.265		
Bilirubin, mg/dL (median, IQR)	0.4 (0.1‐0.3)	0.5 (0.2‐2.3)	0.5 (0.117‐2.37)	0.2 (0.3‐1.4)	0.314		
Albumin, g/dL (median, IQR)	4.1 (3‐42.9)	4.3 (2.9‐43)	4.3 (2.9‐43)	1.0 (1.0‐1.1)	0.008		
INR (median, IQR)	1.2 (0.66‐1.8)	0.9 (0.68‐1.8)	0.91 (0.66‐1.8)	25.0 (10.6‐59.0)	<0.001		
Platelets (×10^9^/L)	206 (39‐451)	229 (14‐504)	226 (14‐504)	1.0 (0.9‐1.0)	0.005		
APRI score (median IQR)	0.63 (0.38‐1.08)	0.51 (0.32‐0.86)	0.53 (0.33‐0.88)	1.00 (0.84‐1.10)	0.773		

^a^ Adjusted OR and *P*‐value only provided for statistically significant factors

### 3.4 Cost per patient treated and per outcome achieved

Resource utilization for the first 492 participants enrolled at the Yangon and Mandalay study sites and locally collected unit costs is presented in Table 4. Table 5 reports the cost per patient treated and per successful outcome produced for the four scenarios modelled. A detailed breakdown by cost component and outcome for scenarios 1 and 2 is provided in Table 6. In Scenario 1, which reflects observed costs minus expenses related to research and represents the likely ‘real‐world’ scenario for the protocol intervention, the treatment cost was an average of $930 per patient; the cost to produce a successful patient, taking into account the cost of unsuccessful outcomes, was $1109. These costs increased to $1129 and $1,216, respectively, when research expenses were included in Scenario 2 (Table 6). Costs were minimized by the removal of routine genotype testing, falling to $980 per patient treated and $1,055 to produce a successful outcome. As might be expected, if the treatment success rate declined, as in Scenario 4, the cost per patient treated remained the same as in Scenario 1, but the cost to produce a successful patient climbed to $1,248 (Table 5).

**TABLE 4:** Median resource utilization per participant during the 24‐week study period

Resource	Median number utilized per patient	CCCost per unit (USD)
Physical examination	3	22.32
Counselling visit	4	5.39
Fibroscan (supplies only)	1	45.23
HCV RNA (Roche quantitative assay)	2	80.00
HCV genotype	1	50.00
Liver tests (ALT, AST, albumin, bilirubin)	2	8.00
CBC (haemoglobin, platelets)	2	5.04
Creatinine	2	5.00
INR/Prothrombin time	2	2.00
HIV screening antibody rapid test	1	1.00
HBV rapid test (Surface Ab, Core Ab, Surface Ag)	1	1.00
SOF/VEL (tablets; mean cost /24 week study period)	84	555.00
Ribavirin (tablets; mean/24 week study period)^a^	123	83.00
Tenofovir cost (mean/24 week study period)	84	33.60

Abbreviations: Ab, antibody; Ag, antigen; ALT, alanine aminotransferase; AST, aspartate aminotransferase; CBC, complete blood count; Hb, haemoglobin; HBV, hepatitis B virus; HCV, hepatitis C virus; HIV, human immunodeficiency virus; INR, international normalized ratio; Ni‐Ni, centralized processing laboratory and current standard of care at the time of study implementation; Plt, platelet count; PT, prothrombin time; RNA, ribonucleic acid; SOF/VEL, sofosbuvir 400 mg/velpatasvir 100 mg.

^a^ 12 patients were prescribed ribavarin at either 400 or 500mg/tablet.

**TABLE 5:** Cost per patient treated and per successful outcome produced, by scenario

Scenario	Cost per patient treated (USD)	Production cost of successful outcome (USD)^a^
1) Research expenses removed	1030	1,109
2) Actual demonstration project as implemented	1129	1,216
3) Routine genotype testing removed	980	1,055
4) Increased treatment failure to 14%	1030	1,248

^a^ Production cost per successful outcome = all costs for cohort/number of successful outcomes.

**TABLE 6:** Cost/patient by outcome in Scenario 1 in USD, for n = 492 included in cost analysis

Outcome^a^	N achieving outcome	Drugs	Laboratories	Events	Indirect costs (Scenario 1)	Indirect costs (Scenario 2)	Mean (SD) cost per outcome (Scenario 1)	Mean (SD) cost per outcome (Scenario 2)
Success	457 (93%)	557	258	113	107	208	1035 (20)	1136 (25)
Failure	17 (3%)	555	264	115	113	218	1047 (20)	1152 (27)
Lost	17 (3%)	555	156	86	79	153	876 (24)	950 (38)
Death	1 (0%)	555	152	84	74	142	864 (N/A)	933
All outcomes	492 (100%)	557	255	112	106	187	1030 (20)	1129 (27)

Abbreviations: SD, standard deviation; USD, United States dollar.

^a^ Only patients at Yangon and Mandaley sites.

## 4 DISCUSSION

In this demonstration project, treatment of HCV among key populations in three regions in Myanmar, including 61.9% PWID, 43.7% HIV co‐infected, and 2.5% HBV co‐infected, utilizing an approach with limited laboratory testing and frequent counselling led to a moderately high SVR rate of 85% amongst all who initiated treatment. Across all sites, virologic failure was demonstrated in 10%, and loss to follow‐up in 5% of participants. Treatment success varied widely by site, with Yangon achieving 96% SVR, Mandalay 83%, and Kachin only 72%. PWID status was associated with treatment failure, and rates of both virologic failure and LTFU were higher in Mandalay and Kachin sites where the majority treated were PWID. While adherence to HCV treatment was not measured, barriers to adherence to treatment amongst PWID may have contributed to low SVR rates. Notably, the SVR rate amongst PWID on OST was significantly higher than for PWID not on OST, suggesting that PWID status alone may not adversely impact treatment success if PWID are linked to substance use and harm reduction treatment. Indeed, other studies have demonstrated high rates of success with HCV treatment co‐located with substance use treatment services and amongst PWID receiving OST.^23^, ^24^

The cost per patient treated estimated in our real‐world scenario, $1030, likely understates the true cost of program implementation, as it excludes costs for scaling up and maintaining the treatment program, such as procurement, training, management and oversight. It also excludes the costs of screening and confirmatory testing for those who do not further engage in care. For budgeting purposes, under the assumption of no genotype testing and decreased real‐world effectiveness but with approximately 20% added for program management, screening and confirmatory testing, an average cost of $1250/patient is likely a reasonable estimate for this intervention in 2019. Although much less than seen in high‐income countries, a cost of $1250/patient will likely be challenging to pay for a country with per capita health expenditures estimated at $62/year (https://knoema.com/atlas/Myanmar/Health‐expenditure‐per‐capita). Nearly half the cost estimated per patient is attributed to the cost of SOF/VEL alone. Reduced drug costs by drug companies in addition to donations by manufacturers and/or foreign donor investments will likely be needed for scale‐up of treatment.

The production cost for a successful outcome reported here is modelled utilizing confirmatory HCV RNA pricing negotiated for the project through Ni‐Ni laboratories. Other confirmatory HCV tests, including those that can be performed at or near the point of care, may allow further reductions in the laboratory cost components, though reliance on foreign imports for parts and servicing may offset cost savings. Other ways to reduce costs include omitting Fibroscan testing, which is not required for an elimination program.

This analysis has several limitations. Given the LTFU and inability to assess SVR in some participants who completed treatment, the true SVR rate is likely slightly underestimated, although the overall LTFU was still somewhat small. While the four cost scenarios presented aimed to provide a reasonable estimate of the costs across a range of strategies in Myanmar, the cost results were based on Yangon and Mandalay treatment outcomes and costs only, excluding those from Kachin. The poorer treatment outcomes and likely higher cost of resource inputs in Kachin, resulting from its more remote location, were not taken into account. Inclusion of rural like Kachin would likely benefit from cost benefit analysis for upscale of simplified HCV treatment protocols.

Several major barriers to universal treatment and elimination of HCV in Myanmar were identified, including proximity to clinics, the cost of DAAs, the cost and feasibility of HCV RNA testing, and significant virologic failure among PWID not on OST. Identification of HCV viremic patients may be improved, with a point of care assay to detect HCV viraemia or antigenemia. Increased access to substance use treatment and harm reduction services is further necessary to improve SVR rates among PWID.

It is critical to appreciate that markers of marginalization such as enforcement‐oriented drug policies, poor patient‐provider relationships, institutionalized stigma within health care systems and social exclusion of PWID are key drivers of challenged treatment compliance and adherence, which impact health seeking behaviour and the ability to access and complete HCV treatment.^25^ PWIDs can achieve adherence to and successful outcomes from HCV treatment comparable to other populations, with low re‐infection rates.^26^, ^27^

A multidisciplinary approach to HCV treatment where treatment and counselling services are offered in a ‘one stop shop’ has been shown to improve treatment uptake and adherence to therapy^28^. This study has shown that given enough governmental and non‐governmental capacity to enrol and treat patients at accessible facilities, a simplified treatment protocol integrated with social and behavioural services, can further increase SVR rates for HCV among all populations, including PWID.^29^

## CONFLICT OF INTEREST

KWC has received a research grant to the institution from Merck Sharp & Dohme.
